# A Hiccup in Hiccup Management: Cardiac Arrest from Previously Undiagnosed Congenital Long QT Syndrome

**DOI:** 10.1155/2018/5023954

**Published:** 2018-10-10

**Authors:** Robert Hughes, Johnathan M. Sheele

**Affiliations:** Department of Emergency Medicine, University Hospitals Cleveland Medical Center & Case Western Reserve University, Cleveland, OH 44106, USA

## Abstract

We report the case of a person who went into cardiac arrest after being given chlorpromazine for hiccups and was subsequently diagnosed with congenital Long QT Syndrome. Long QT Syndrome is an uncommon, congenital condition that carries a high risk of sudden cardiac death. Clinicians need to recognize the risk that chlorpromazine may prolong the QTc and prepare to manage potential complications.

## 1. Background

Singultus (hiccups) are a common and usually short-lived condition. Hiccup bouts are usually self-limited and last < 48 hours. Persistent hiccups last >48 hours, and intractable hiccups last greater than two months [1, 2]. Prolonged bouts of hiccups can produce significant morbidity and are most effectively managed by treating the underlying condition if it can be identified in the emergency department (ED). There are many causes of hiccups including gastric distention, aerophagia, ventriculoperitoneal shunts, hydrocephalus, multiple sclerosis, strokes, epidural or subdural hematomas, diffuse axonal injury, cerebral contusions, encephalitis, meningitis, brain abscesses, neurosyphilis, laryngitis, pharyngitis, irritation of the tympanic membrane, retropharyngeal abscess, peritonsillar abscesses, mediastinitis, esophagitis, bronchitis, pneumonia, myocardial infarction, pericarditis, aortic aneurysms, pericarditis, misplaced pacemaker wires, small bowel obstruction, perihepatitis, subphrenic abscess, goiter, tumor or cyst of the neck, barbiturates, steroids, methyldopa, and electrolyte abnormalities [1–4]. Chlorpromazine (Thorazine), a phenothiazine type first-generation antipsychotic drug, is likely the most effective and well-studied pharmaceutical drug for hiccups [1, 2]. Chlorpromazine has several potential adverse effects including sedation and the potential to prolong the cardiac QTc [1, 2].

## 2. Case Report

A 48-year-old Caucasian male with a past medical history of hypertension, diverticulosis, bicuspid aortic valve with mild insufficiency, and daily alcohol use presented to the emergency department (ED) with 1 week of hiccups associated with a few episodes of nonbloody and nonbilious emesis with no chest pain, abdominal pain, or dyspnea. He was not taking any medications. He had no known prior history of palpitations, syncope or seizures. There was no known significant family medical history. His triage vital signs were: temperature of 36.2°C; blood pressure of 140/90 mmHg; heart rate of 114; respiratory rate of 18; and an oxygen saturation of 96% on room air. Physical examination showed dry mucus membranes, regular heart rate without murmur, soft abdomen, and active hiccups. The patient was given a 1-liter bolus of intravenous normal saline and chlorpromazine (Thorazine) 25 mg by mouth. Approximately 15-20 minutes after receiving chlorpromazine, the patient was found in cardiac arrest, and advanced cardiac life support (ACLS) interventions were initiated. The initial rhythm was ventricular fibrillation, and he received a total of four defibrillations at 200 joules each. Torsades de pointes was also observed, and he received 2 grams of intravenous magnesium.

**Figure 1:**
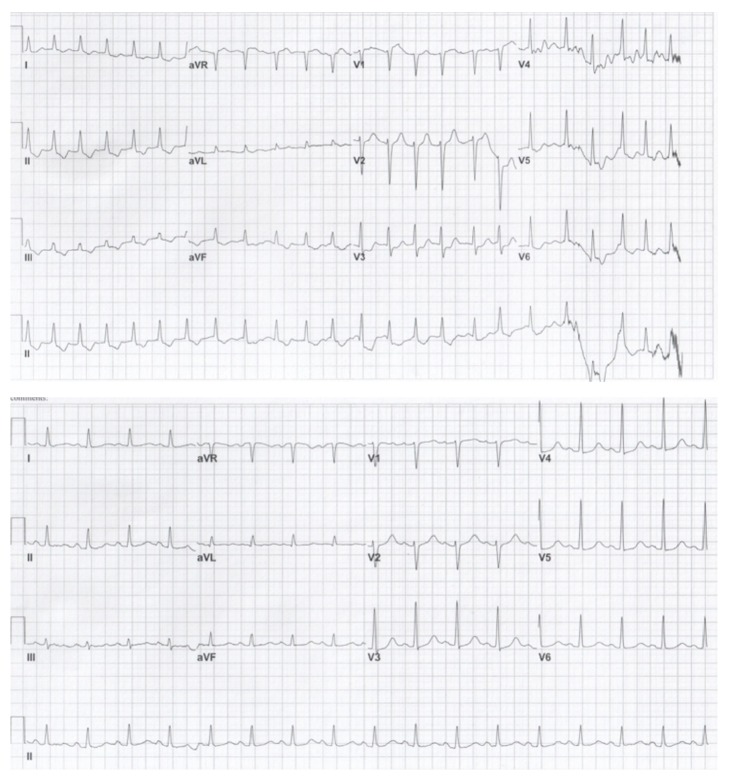
EKGs obtained after the patient experienced cardiac arrest demonstrated a prolonged QTc.

The post-arrest ECGs (Figure 1) were notable for normal sinus rhythm with a rate of 98-99, and a QTc of 495-521 ms (normal QTc is <440 ms in men). Immediate post-return of spontaneous circulation (ROSC) arterial blood gas was significant for metabolic acidosis with a pH of 7.22 (range, 7.38–7.42), a lactate of 14 mmol/L (range, 0.60–2.40), and an ionized calcium of 1.12 mmol/L (range, 1.10–1.33). A comprehensive metabolic panel was remarkable for a glucose of 206 mg/dL (range, 74–99), potassium of 3.0 mmol/L (range, 3.5–5.3), bicarbonate of 16 mmol/L (range, 21–32), an anion gap of 30 mmol/L (range, 10–20), aspartate transaminase (AST) of 466 U/L (range, 10–37), alanine transaminase of 374 U/L (range, 10–65), and serum total bilirubin of 1.9 mg/dL (range, 0.0–1.2). A complete blood count was only significant for a platelet count of 101 x 10E9/L (range, 150–450). Blood and urine drug screens, thyroid stimulating hormone level, magnesium level, and a cardiac troponin, were all found to be normal.

**Figure 2:**
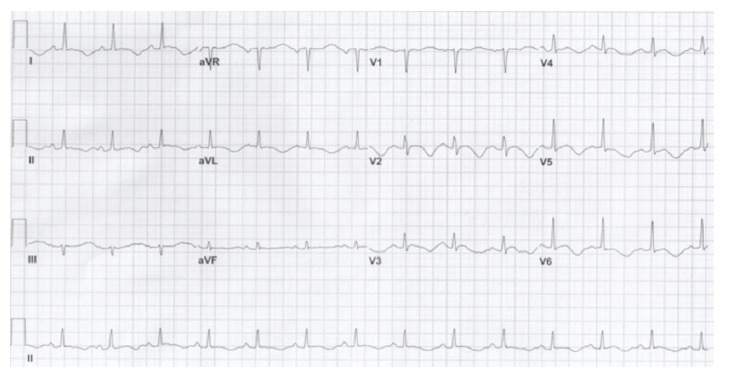
EKG on hospital day 11 with a QTc of 611 ms with a serum potassium level of 3.8 mEq/L and a magnesium level of 2.1 mEq/L.

Chest X-ray, computed tomography of the head, chest, abdomen, and pelvis were unremarkable. Post-ROSC echocardiogram showed diffuse areas of hypokinesis and an ejection fraction (EF) of 20-25% which improved a week later to no wall motion abnormalities and an EF of 60-65%. Cardiac troponin peaked at 0.48 ng/mL (range, 0.00–0.06), and a cardiac catheterization showed no coronary artery disease. Multiple post-arrest ECGs consistently demonstrated a prolonged QTc with measurements up to 611 ms despite optimization of electrolytes (Figure 2). An old ECG from a past clinic visit showed a QTc of 581 ms. Electrophysiology diagnosed him with congenital Long QT Syndrome for which he received a dual chamber implantable cardioverter defibrillator (ICD). The patient was extubated on day 6, transferred from the intensive care unit to the medical floor on day 12, and discharged from the hospital on day 19. It was not clear what precipitated the patient's hiccups.

## 3. Discussion

Prolonged QTc on ECG, particularly >500 ms, can be congenital or acquired. It can be the result of hypokalemia, hypocalcemia, hypomagnesemia, hypothermia, myocardial ischemia, drugs (including antimicrobials, antiarrhythmics, and antipsychotics), increased intracranial pressure, post-cardiac arrest, and via inherited Long QT Syndrome [5]. Long QT Syndrome is a manifestation of a collection of heritable ion-channel mutations with an estimated prevalence of about 1:2000 in the population [6]. Two phenotypes of Long QT Syndrome include Romano-Ward and Jervell Lange-Nielsen syndromes. Romano-Ward Syndrome is the more benign variant with severity ranging from asymptomatic and undiagnosed to sudden cardiac death [7]. Jervell Lange-Nielsen Syndrome is the more severe form; it includes sensorineural hearing loss and may require genetic testing for diagnosis [8].

Mechanism of the prolongation and clinical manifestations are due to alterations in the rectifying potassium and sodium channels in cardiac myocytes. This may be directly due to toxicity of the channel, alterations in electrolytes, or congenital mutations of the channels themselves [9, 10]. Categorization of the congenital mutations in Romano-Ward Syndrome is based on a numbering scheme with several mutations identified with types 1-3 being the most common forms seen. LQT1 and LQT2 encode a rectifying potassium channel, while LQT3 encodes the alpha Sodium channel subunit. The mutations of the channel directly, or a regulatory subunit, directly alters the ionic current and membrane potential of the myocyte, leading to the characteristic arrhythmia, polymorphic ventricular tachycardia [7].

An important distinction to be made between congenital and acquired is the ability to correct the abnormality by addressing the underlying disorder (i.e., electrolyte abnormality) or discontinuation of the offending drug. As our patient demonstrated, the QT prolongation persisted despite correction of electrolyte deficiencies. Taking this in to consideration, treatment varies based on etiology. First line for acquired etiologies is to address the inciting event, be it drug, electrolyte imbalance, etc. Congenital etiologies usually require an ICD placement for early defibrillation of malignant rhythms [11, 12]. Further management with beta-blockade suppresses sympathetic activity [11, 12]. Some polymorphisms respond differently to beta-blockers so genetic testing can help with diagnosis, prognostication, and treatment [13, 14]. Further risk mitigation is provided with patient counseling about medications and nutrition supplements that may prolong the QTc and recommendation to avoid these, as well as advising caution with participation in competitive sports.

Several drugs, including chlorpromazine, are known to prolong the cardiac QTc although the precipitation of cardiac arrhythmias is rare. Clinicians providing intravenous chlorpromazine need to be aware that the drug can prolong the cardiac QTc it may be prudent to evaluate the QTc prior to medication administration, and to be aware of potential complications.
